# Functional Divergence of the Paralog *Salmonella* Effector Proteins SopD and SopD2 and Their Contributions to Infection

**DOI:** 10.3390/ijms25084191

**Published:** 2024-04-10

**Authors:** Mosopefoluwa T. Oke, Vanessa M. D’Costa

**Affiliations:** 1 Department of Biochemistry, Microbiology and Immunology, University of Ottawa, Ottawa, ON K1H 8M5, Canada; 2 Centre for Infection, Immunity and Inflammation, University of Ottawa, Ottawa, ON K1H 8M5, Canada

**Keywords:** bacterial pathogenesis, intracellular pathogen, intracellular trafficking, host–pathogen interactions, *Salmonella enterica*, effector protein, Rab GTPase

## Abstract

*Salmonella enterica* is a leading cause of bacterial food-borne illness in humans and is responsible for millions of cases annually. A critical strategy for the survival of this pathogen is the translocation of bacterial virulence factors termed effectors into host cells, which primarily function via protein–protein interactions with host proteins. The *Salmonella* genome encodes several paralogous effectors believed to have arisen from duplication events throughout the course of evolution. These paralogs can share structural similarities and enzymatic activities but have also demonstrated divergence in host cell targets or interaction partners and contributions to the intracellular lifecycle of *Salmonella*. The paralog effectors SopD and SopD2 share 63% amino acid sequence similarity and extensive structural homology yet have demonstrated divergence in secretion kinetics, intracellular localization, host targets, and roles in infection. SopD and SopD2 target host Rab GTPases, which represent critical regulators of intracellular trafficking that mediate diverse cellular functions. While SopD and SopD2 both manipulate Rab function, these paralogs display differences in Rab specificity, and the effectors have also evolved multiple mechanisms of action for GTPase manipulation. Here, we highlight this intriguing pair of paralog effectors in the context of host–pathogen interactions and discuss how this research has presented valuable insights into effector evolution.

## 1. Introduction

The species *Salmonella enterica* (*Salmonella*) comprises over 2600 recognized serovars that can infect diverse eukaryotic species [1]. As human pathogens, these serovars are broadly classified as either non-typhoidal or typhoidal, with the typhoidal serovars (i.e., Typhi, Paratyphi A, B, and C) being host-restricted to humans [1,2,3]. *Salmonella* is most often transmitted via the ingestion of contaminated materials [1,2,3]. Typhoidal serovars are responsible for enteric fever (or typhoid fever), while disease by non-typhoidal serovars typically presents as gastroenteritis, which can become invasive and extraintestinal in at-risk populations like the elderly and immunocompromised [1,2,3]. As one of the leading agents of acute bacterial gastroenteritis, Salmonellae are responsible for a third of the deaths associated with food-borne diseases [1], and non-typhoidal serovars cause approximately 93 million illnesses annually [4]. 

One of the hallmarks of *Salmonella* infection is bacterial-mediated entry into non-phagocytic cells and intracellular survival in multiple host cell types within a bacteria-containing vacuole termed the *Salmonella*-containing vacuole (SCV) [5,6]. SCVs are described as late-endosome-like compartments marked by the presence of late endosome/lysosomal-associated membrane proteins such as LAMP1 and LAMP2 [6,7]. Bacterial entry and survival in non-phagocytic host cells are facilitated by virulence proteins termed effectors, which are translocated into host cells via two independent Type III secretion systems (T3SSs) [8,9,10]. These two distinct T3SSs are encoded on two horizontally acquired islets termed *Salmonella* pathogenicity islands 1 and 2 (SPI-1 and SPI-2, respectively) [10]. 

Translocated into host cells through specialized secretion systems, bacterial effector proteins typically mediate pathogenesis by targeting host proteins or protein complexes to manipulate host cellular pathways for the benefit of the bacteria [9,11]. In the context of *Salmonella* infection, effector proteins play an instrumental role in pathogenesis, functioning by modulating or hijacking host cellular processes to contribute to survival, replication, and cell-to-cell spread [9,12]. The effectors secreted by the SPI-1 T3SS are responsible for bacterial entry into non-phagocytic cells and promote early biogenesis of the SCV [13], while effectors of the SPI-2 T3SS contribute to the evasion of cellular immune responses to infection [14], bacterial replication [15,16], and systemic spread [17]. Between the two T3SSs, over 40 bacterial effectors with diverse host targets and mechanisms are secreted into host cells [12,18]. During the course of evolution, the host specificity and type of disease caused by the different *Salmonella* serovars are believed to have resulted in selective pressures that altered the repertoire of secreted effectors [19], with certain effectors remaining highly conserved in the genome and others either absent or nonfunctional [18,19,20]. 

The *Salmonella* genome encodes several sets of effectors that share high levels of sequence similarity [21]. These sets of effector genes are considered paralogs, believed to have arisen from gene duplication in an ancestral *Salmonella* lineage [21]. Examples of paralog effectors include Pathogenicity island-encoded protein B (PipB) and PipB2, which share 33% identity and 67% similarity [22]; *Salmonella*-induced filament proteins A (SifA) and SifB that share 26% identity and 46% similarity [23]; *Salmonella* outer protein E (SopE) and SopE2, which share 69% similarity [24]; and *Salmonella* outer protein D (SopD) and SopD2 that share 43% identity and 63% similarity [21]. These effector protein paralogs often share structural similarity and/or biochemical activities but demonstrate functional divergence in intracellular localization and/or host protein targets or interaction partners [12]. 

The effector proteins SopD and SopD2 are among the more well-studied effector paralogs at the level of molecular mechanism and represent an interesting example of functional divergence. The paralog effectors have evolved to be secreted by different T3SSs [25], and the effectors have demonstrated divergence in intracellular localization properties [26], host targets [27], and contributions to infection [28,29]. This review will focus on the evolution of these two effector proteins, highlighting established functional similarities and differences and overall contributions to *Salmonella* pathogenesis. 

## 2. SopD and SopD2: *Salmonella enterica* Paralog Effectors

### 2.1. Discovery of SopD and Early Insights into Function

The gene encoding SopD was first described in 1989 as part of a sequencing study of an adjacent operon (*cysJIH*) in *S*. Typhimurium LB5000 [30]. Originally cited as an open reading frame of unknown function (*orf*-*4*) [30], the gene was renamed *sopD* in 1998 after the characterization of its homolog in *S*. Dublin [31]. The *sopD* gene is located in the genome of *S*. Typhimurium in centisome 64 [31], downstream of SPI-1 [20]. The gene is highly conserved across the *Salmonella* genus, and it is present in both species of *Salmonella*, *S. enterica*, and *S. bongori* [20,31,32]. 

SopD can be secreted during the early stages of infection by the SPI-1 T3SS [25,31]. In vitro, secretion has been observed under SPI-1- and SPI-2- T3SS-inducing conditions, with stronger secretion levels observed in SPI-1-inducing conditions [25]. It is essential for virulence in mouse and calf models of infection [21,33,34,35] and was shown to contribute to enteropathogenicity and diarrhea in calf models [31]. Additionally, it contributes to the intracellular replication of *Salmonella* in macrophages [21]. 

Among the first functional insights, studies with wild-type *S*. Dublin and an isogenic *sopD* insertional inactivation mutant in a bovine ligated ileal loop infection model suggested that the effector contributes to fluid secretion and polymorphonuclear leucocyte influx through an unknown mechanism, an effect that was additive with a mutant of the SPI-1 T3SS-secreted effector SopB [31]. SopD was also shown to contribute to bacterial invasion of polarized epithelial cells, a model representative of the cells lining the gastrointestinal system [36]. 

In host cells, SopD displays partial localization with early and late endosomes and with *Salmonella* at the bacterial invasion sites [26]. The invasion of non-phagocytic cells by *Salmonella* is a dynamic process that involves the actions of multiple effectors of the SPI-1 T3SS on the actin cytoskeleton [37]. The recruitment of SopD to the invasion site is promoted by the lipid phosphatase activity of SopB [38], which mediates the dephosphorylation of plasma membrane lipid phosphatidylinositol-4,5-bisphosphate (PI(4,5)P_2_) [26,39]. In the context of cell biological studies, the membrane-binding ability of SopD is impaired by fixation with paraformaldehyde [26]. Both SopD and SopB contribute to promoting plasma membrane sealing and scission to result in the formation of the SCVs [26,39].

### 2.2. SopD2: A Paralog Effector in S. enterica

The effector SopD2 was first described in 2003 [25]. Identified from a genome mining analysis of the recently sequenced strain *S*. Typhimurium LT2 [40], *sopD2* is encoded in centisome 19.5 [25]. Present on an islet with a lower GC content than the majority of the *Salmonella* genome [25] and absent in *S. bongori* [41], *sopD2* is believed to have arisen through genetic duplication and subsequent divergence from an ancestral version of *sopD* [25]. For *sopD2*, orthologous sequences are present in diverse serovars of *S. enterica* [18,42]. The gene is considered to be widely conserved, especially among serovars commonly associated with gastrointestinal diseases [18,43]. Notably, it is a pseudogene in the human-adapted agents of enteric fever, *S*. Typhi and *S*. Paratyphi A [18,42,43]. 

Unlike SopD, SopD2 is secreted into host cells by the SPI-2 T3SS but not the SPI-1 T3SS, and in epithelial cells, secretion is not detected until at least 5 h post-infection [25]. Like SopD, SopD2 also contributes to the virulence of *Salmonella* in mouse models and contributes to replication in macrophages [21,25,34,44]. A systematic analysis of *S*. Typhimurium knockout mutants defined *sopD2* as part of the minimal effector repertoire required for SCV division and bacterial replication [16].

Functionally, the deletion of *sopD2* in the *S*. Typhimurium genome has been shown to affect *Salmonella*-induced tubule (SIT) formation [45,46,47,48], a phenotype characteristic of *Salmonella* infection associated with an extensive remodeling of the endosomal system resulting in the formation of a network of intracellular single- and double-membrane tubules extending along host microtubules that are marked by the presence of one of several combinations of host and/or bacterial proteins [46]. While their role during infection is not fully understood, SITs have been proposed to contribute to nutrient acquisition and cell-to-cell spread [49,50,51]. SopD2 has been shown to contribute to the formation and extension of LAMP1-positive *Salmonella*-induced filaments (SIFs) [15,21,25], *Salmonella*-induced secretory carrier membrane protein 3 (SCAMP3)-positive tubules (SISTs) [47], and inter-cell tubules (ICTs) [48], but it inhibits the formation of LAMP1-negative tubules (LNTs) [45]. 

SopD2 has also been shown to contribute to the suppression of antigen presentation in dendritic cells, a SPI-2 T3SS-dependent phenotype [14]. This phenotype has been suggested to occur at the level of intracellular trafficking along microtubules, where processed peptides are delivered to major histocompatibility complex class II (MHC-II) complex-containing compartments for subsequent peptide loading [14]. SopD2 may also contribute to the bacterial-mediated suppression of the nuclear translocation of the host transcription factor EB (TFEB), a protein that promotes the transcription of lysosomal- and autophagy-related genes [52,53], in the early response to bacterial phagocytosis by macrophages [54]. However, a direct contribution of SopD2 to this phenotype in macrophages and any associated host targets and mechanism of action are unknown [54]. 

The SCV represents the primary intracellular niche for *Salmonella*, and the stability of this vacuole is maintained by several effectors of the SPI-2 T3SS [15,16,45]. SopD2 has also been suggested to modulate SCV membrane stability [45]. During SCV maturation, the vacuole traffics towards the microtubule organizing center (MTOC) and the SCV anchors to the Golgi apparatus [6,7]. The deletion of *sopD2* in *Salmonella* causes the bacteria to lose perinuclear localization [15], suggesting that SopD2 contributes to maintaining SCV intracellular positioning. In the context of SCV morphology, most intracellular bacteria are housed in individual SCVs, such that, during replication, each daughter cell is present in a separate SCV [16,55]. SopD2, along with the effectors SteA and SifA, was shown to contribute to the proper division of SCV membranes during bacterial replication [16]. 

At the level of intracellular localization, SopD2 localizes to late endosomes and lysosomes in host cells and can be found on the SCV membrane and SIFs in infected cells [21,25,56]. Given this targeting to late endosomes and lysosomes, it demonstrates partial colocalization with its paralog effector SopD [25]. Unlike SopD, the membrane-binding ability of SopD2 is not impaired by paraformaldehyde fixation [25].

### 2.3. SopD and SopD2: Structure–Function Relationships

**Figure 1:**
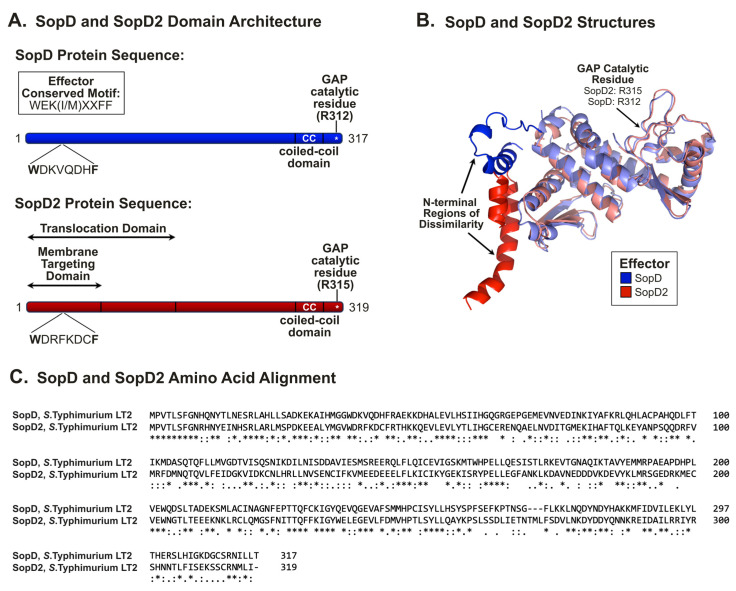
**The *Salmonella* effectors SopD and SopD2: Domain architecture, structure, and amino acid alignment.** (**A**) Primary amino acid sequence of SopD (blue) and SopD2 (red), where amino acid numbering is indicated. Sites of importance are denoted, where applicable. (**B**) Three-dimensional structure of SopD (blue) and SopD2 (red), shown as an overlay [28]. Information regarding modeling and superimposition is provided in [28]. (**C**) Amino acid alignment of effectors from *S.* Typhimurium LT2. Accession numbers for SopD and SopD2 are NP_461866 and NP_459947, respectively. ClustalW alignment is shown with identical (*), highly conserved (:), and conserved residues (.) denoted.

With respect to primary amino acid sequence, SopD and SopD2 have similar lengths (317 and 319 amino acids, respectively) (Figure 1A), and the effectors demonstrate sequence similarity throughout the entirety of the peptides [25]. Both proteins contain a *Salmonella* effector translocation signaling motif in their N-terminal region, termed the W(E/D)(K/R)xxxF motif, present between amino acids 37 and 44 that is essential for effector translocation [56], and both effectors possess coiled-coil domains [22,25,26] (Figure 1A). The localization of SopD to intracellular vesicles has been shown to require both the N-terminal effector-targeting motif and the C-terminal coiled-coil domain [26]. Conversely, for SopD2, the first 75 amino acids were demonstrated to be sufficient for the appropriate targeting to membranes, and the first 200 amino acids were shown to be sufficient for translocation through the SPI-2 T3SS [25]. It was also observed that the mutation of the coiled-coil domain (SopD2^Y281D,Y284D,K288D^) results in a loss of membrane association, suggesting that this domain might also contribute [57]. 

From the perspective of three-dimensional structures, SopD and SopD2 demonstrate structural similarity throughout the majority of the peptide sequence (Figure 1B) [28]. The most notable region of dissimilarity was observed in the amino-terminal regions, where each effector occupies a unique three-dimensional space [28]. With a 43% sequence identity at the amino acid level, the level of sequence similarity is relatively consistent throughout the entirety of the peptides (Figure 1C).

## 3. SopD and SopD2: Host Interactors and Molecular Mechanisms of Action

### 3.1. Host Cell Interaction Partners 

Bacterial effectors typically function by initiating protein–protein interactions with a host protein or protein complex [9]. To date, several studies have identified host interaction partners of SopD and SopD2 (Table 1). Both SopD and SopD2 have demonstrated an affinity for binding host Rab GTPases [27,28,29,58,59,60]. Validated Rab interactors for SopD include Rab8A and Rab8B [27,60], Rab10 [27,29], Rab14 [27], and Rab3D [27], while SopD2 has been shown to be capable of interacting with Rab7A [31], Rab32 [27,58], Rab34 [59], Rab8A and Rab8B [58,60], Rab10 [29,58], Rab29 [27], and Rab38 [27].

Outside regulatory GTPases, additional host interaction partners for the effectors have been identified (Table 1). For SopD, proteins involved in microtubule function (MAP1B, CKAP5, DCTN2, DYNC1H1, KIF5B, KIF11), retromer complexes (VPS35, VPS26A), actin function (MYO5B), and adaptor–protein complexes (AP3S1, SCYL2, CLTC, AP2M1) have been detected [29]. 

For SopD2, established interactors include annexin A2 (AnxA2) [62] as well as proteins involved in actin function (MYH10, MYL6, MYL12B, MYH9, CYFIP1, MYO1B), RNA binding and translation (RBM10, EIF3B, EIF3A, EIF3E), and adaptor–protein complex (AP2B1, AP3D1, AP3B1, AP2A1) [61]. While the consequences of many of these host–pathogen protein–protein interactions are not fully understood, several of the host interactors have been characterized as targets of SopD and/or SopD2.

### 3.2. Host Cell Targets and Effector-Mediated Manipulation of Host Function

Bacterial effectors have evolved a diverse array of mechanisms of action on host cells to mediate infection [9,11]. Among the many molecular strategies, effectors can functionally mimic host proteins or can activate or suppress host pathways [9,11]. Effectors also often demonstrate multifunctionality, harboring multiple domains within the same protein that mediate unique biological functions [11]. SopD and SopD2 have evolved to target members of a family of regulatory proteins called Rab GTPases using two distinct mechanisms [28,29,58,60]. Cell biological studies have also suggested that they may be capable of targeting other host cellular processes as well [62]. 

#### 3.2.1. SopD2: Rab GTPase Targets

Rab GTPases (Rabs) are a large family of regulatory proteins in eukaryotes that function as molecular switches to control diverse aspects of vesicular trafficking, including the biogenesis, transport, tethering, and fusion of membrane-bound organelles and vesicles [63,64]. Considering their vital role in endosome, phagosome, and lysosome trafficking and maturation, and that vacuolar pathogens must evolve mechanisms to direct their own trafficking within host cells, Rab GTPases are often targeted by intracellular vacuolar pathogens, including *Salmonella* [65,66].

Each Rab GTPase functions by localizing to distinct membrane-bound intracellular compartments and mediating trafficking via interaction with binding partners termed Rab-effectors [63,64]. The functionality of Rabs depends on their ability to cycle between inactive GDP-bound and active GTP-bound conformations, a cycle that is regulated by proteins called guanosine nucleotide exchange factors (GEFs) and GTPase-activating proteins (GAPs) [63,64]. GEFs exchange GDP for GTP, thereby activating the Rabs, while GAPs catalyze the hydrolysis of GTP to GDP, thus inactivating the Rabs [63,64]. 

**Figure 2:**
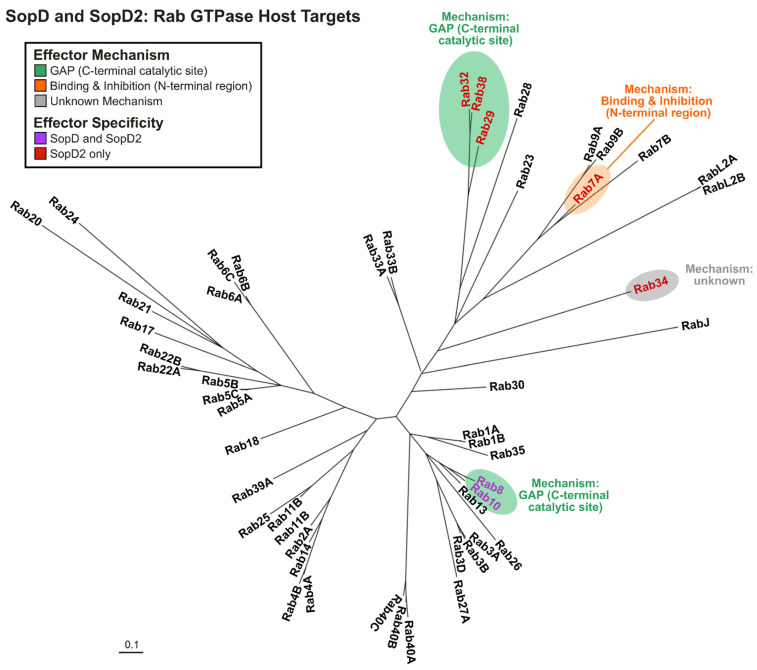
**SopD and SopD2: Rab GTPase host targets.** Phylogenetic tree of human Rab GTPase proteins. Rabs known to be targeted by both effectors are labeled in purple, and Rabs shown to be targets of SopD2 only are in red. Effector-mediated Rab suppression by the C-terminal GAP function is highlighted in green, whereas suppression by the N-terminal region of SopD2 is in orange. Where the mechanism is unknown, the GTPase is highlighted in grey. Rab8A and Rab8B are collectively designated as Rab8.

SopD2 was the first effector to be studied in the context of GTPase function. Among the poorly understood hallmarks of *Salmonella* infection has been the pathogen’s ability to globally suppress host endocytic trafficking to lysosomes as a mechanism of host evasion [67]. While the phenotype was previously established [67], the host target, the effector responsible, and its mechanism of action were yet to be established. Through a screen of effector knockout *Salmonella* strains, SopD2 was identified and subsequently shown to be both necessary and sufficient to suppress global endocytic trafficking to lysosomes [28]. The GTPase Rab7A represents a central regulator in the endocytic pathway, facilitating the maturation of early endosomes to late endosomes and the fusion of late endosomes with lysosomes [68]. SopD2 was shown to directly interact with Rab7A to lock Rab7A in the GDP-bound state by suppressing GDP to GTP nucleotide exchange [28]. Through suppression of the formation of active Rab7A, SopD2 was shown to impair the recruitment of Rab7A effectors RILP and FYCO1 [28], responsible for mediating trafficking by conjugating Rab7A-positive compartments to the microtubule apparatus [69,70]. Through a series of domain mapping studies, this function was shown to be specific for SopD2, as SopD could not induce this phenotype, and was functionally mapped to the N-terminal region of SopD2 (Table 1 and Figure 2), consistent with the observed structural divergence at the amino terminus (Figure 1B) [28]. SopD2-mediated suppression of Rab7A trafficking is proposed to contribute to the previously observed phenotypes associated with SCV stability [45] and the suppression of antigen presentation in dendritic cells [14], both established microtubule-associated phenotypes [14,45]. This mechanism of action is also consistent with the phenotype of modulating SCV positioning [15].

In the context of effector multifunctionality, SopD2 has also been shown to mediate a second distinct mechanism of targeting Rab GTPases [58]. Rab32 mediates a cell-autonomous defense mechanism against intracellular pathogens like *Salmonella* [58,71], *Listeria monocytogenes* [72], and possibly *Mycobacterium tuberculosis* [73] via the delivery of itaconate to the bacteria-containing vacuole at growth-inhibiting concentrations [74]. SopD2 has been shown to target and suppress Rab32 function in concert with another *S*. Typhimurium effector GtgE [58]. The Rab32 pathway is believed to serve as an important cell response pathway to bacterial infection in vertebrates, especially in cells of hematopoietic origins [58,71,74], and the absence of *gtgE* [18,42] and pseudogenization of *sopD2* [43] in *S*. Typhi are proposed to result in a restriction in intra-host niche in humans relative to the more broad host serovars like Typhimurium and Enteritidis [58]. Functionally, SopD2 deactivates Rab32 by acting as a GAP via the catalytic arginine residue in its C-terminus, R315 (Table 1 and Figure 1) [27,58], and GtgE preferentially cleaves the inactivated Rab32 [75]. Collectively, this is believed to result in the complete inactivation of Rab32 in host cells to promote bacterial replication and survival [71,75]. With respect to effector specificity, the inactivation of Rab32 has been shown to be specific for SopD2, as in vitro GAP assays demonstrated that SopD2, but not SopD, could inactivate Rab32 and the closely related Rab29 and Rab38 (Table 1 and Figure 2) [27]. However, the consequences of SopD2-mediated Rab29 and Rab38 inactivation on infection have yet to be elucidated.

In addition to these GTPases, SopD2 has also been shown to inactivate Rab8A and Rab10 through in vitro GAP assays [27,58]. However, unlike the substrates in the Rab32 sublineage, Rab8A and Rab10 were also demonstrated to be inactivated by SopD (Table 1 and Figure 2) [27]. Rab8A and Rab10 have yet to be studied in the context of SopD2 during infection.

Another GTPase implicated with SopD2 function is Rab34 [59]. Rab34 is a GTPase that localizes predominantly to the Golgi apparatus [76] and has been linked to the modulation of lysosomal positioning [76], membrane ruffling and macropinosome formation [77], and regulation of constitutive secretion [78] in host cells via interactions with specific Rab-effectors. Identified from immunoprecipitation coupled with mass spectrometry (IP-MS) assays using GFP-tagged SopD2, the interaction with Rab34 was shown to be specific for SopD2, as the GTPase was not detected in comparison experiments with GFP-SopD or GFP (Table 1, Figure 2) [59]. Intracellular survival assays in epithelial cells suggested that Rab34 may contribute to bacterial replication [59]. While Rab34 was shown to directly interact with SopD2 in vitro [59], the mechanism of action has yet to be elucidated.

#### 3.2.2. SopD: Rab GTPase Targets

In addition to interacting with Rab GTPases, SopD has been implicated in manipulating GTPase function [27,29,60]. The first evidence of Rab GTPase modulation by SopD was published in 2021, several years after the first studies on SopD2 [27,29,60]. *Salmonella* infection induces a T3SS-dependent release of pro-inflammatory cytokines, and SopD has been shown to contribute to this process by targeting Rab8A and Rab8B using its C-terminal GAP site (Table 1 and Figure 2) [60]. In the context of infection, Rab8A was observed to be recruited to the *Salmonella* invasion site, and SopD was shown to promote the upregulation and downregulation of pro- and anti-inflammatory cytokines, respectively [60]. Interestingly, at the level of binding, crystal structure analysis of SopD in a complex with Rab8A highlighted a residue in SopD, Glu293, which is not present in SopD2, that contributes to complex formation [60]. Mechanistically, SopD can also modulate Rab GTPase function independently of the C-terminal GAP site [60]. SopD was shown to promote the activation of Rab8 by displacing the GTPase from its guanosine dissociation inhibitor (GDI), releasing it for membrane association and GTP-loading, and an activity that was independent of the GAP site [60].

**Figure 3:**
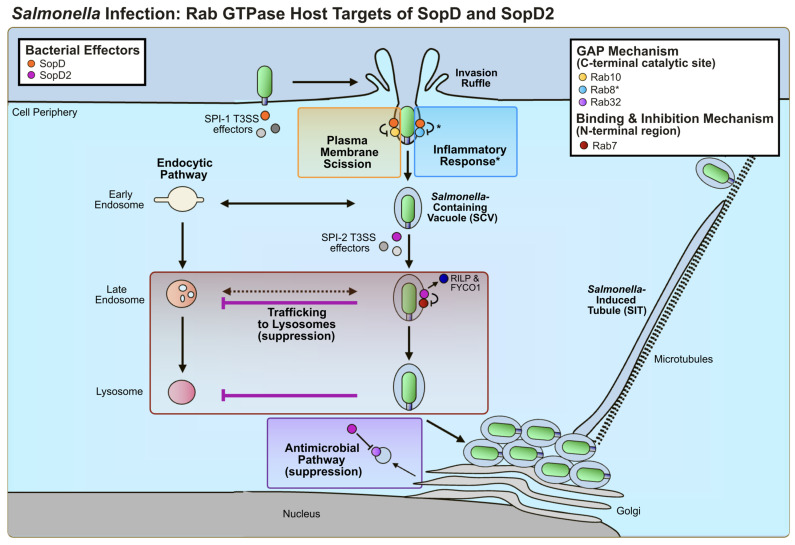
**SopD and SopD2: Molecular mechanisms of action against host Rab GTPases.** The effector SopD (shown in orange) is secreted by the SPI-1 T3SS during early infection of nonphagocytic host cells, and it has been shown to functionally target Rab10 (yellow) and Rab8 (light blue) using its C-terminal GAP site. The modulation of Rab10 results in plasma membrane scission to complete the formation of an intact *Salmonella*-containing vacuole (SCV). In the context of Rab8, the GAP activity of SopD results in the inhibition of anti-inflammatory signaling, whereas its GDI-displacement activity results in the stimulation of anti-inflammatory signaling. The asterisk (*) denotes this bifunctional nature of SopD in targeting Rab8. SopD2 (shown in pink) is secreted by the SPI-2 T3SS after uptake into the SCV, and has been shown to functionally target Rab32 (purple) and Rab7A (Rab7, red). In the context of Rab32, the GAP activity of SopD2 results in the suppression of a cell-autonomous defense mechanism resulting in the delivery of itaconate. Conversely, SopD2 uses its N-terminal region to bind and suppress Rab7. Rab7 targeting by SopD2 prevents the recruitment of the endogenous trafficking-related Rab7 effectors RILP and FYCO1 to the SCV, allowing *Salmonella* to suppress SCV trafficking to lysosomes.

SopD was also shown to target Rab10 during invasion through its C-terminal catalytic arginine residue R312 (Table 1 and Figure 2) [27,29]. SopD is known to contribute to the fission of the bacteria-containing vesicles during invasion, and this activity has been linked to its cooperation with SopB [26,39]. During invasion, Rab10 was observed to be recruited to actin-rich sites at the plasma membrane, promoting the recruitment of its Rab-effectors MICAL-L1 and EHBP1 [29]. SopD-mediated suppression of Rab10 was shown to promote the recruitment of Dynamin-2 to drive plasma membrane scission, resulting in the formation of an intact SCV membrane [29]. A model highlighting the characterized molecular mechanisms of these paralog effectors against Rab GTPases is shown in Figure 3.

#### 3.2.3. Other Host Targets

To date, the majority of characterized host targets of SopD and SopD2 have been Rab GTPases. However, a recent study highlighted the potential for a non-GTPase host target for SopD2. Using stable isotope labeling of amino acids in cell culture (SILAC) in an infection-based assay of epithelial cells, the host protein annexin A2 (AnxA2) was identified as a candidate interactor of both SopD2 and SPI-2 T3SS effector PipB2 (Table 1) [62]. Given that SopD2 was previously implicated in SCV perinuclear positioning [15], a possible role of AnxA2 in this phenotype was further investigated. Further studies demonstrated that the shRNA-mediated suppression of AnxA2 resulted in an impairment in SCV perinuclear positioning, suggesting that AnxA2 may contribute to this infection phenotype [62]. While the molecular mechanism has yet to be determined, it will be interesting to determine in future studies whether this phenotype is independent of the SopD2-mediated Rab7A suppression that was previously shown to modulate microtubule-based trafficking [28].

## 4. Discussion

**Figure 4:**
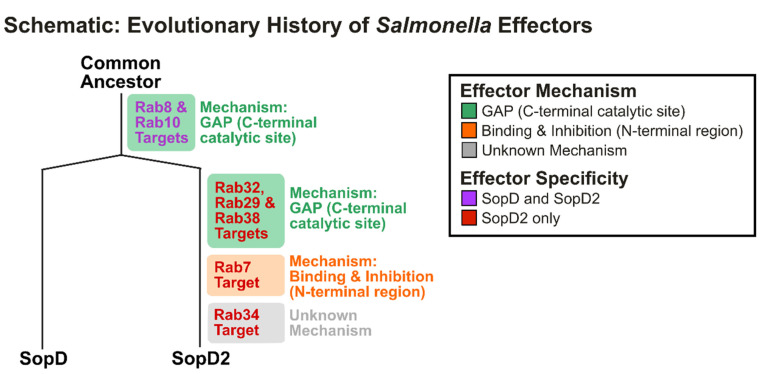
**SopD and SopD2: Proposed model of effector evolution.** Several Rab GTPases have been validated as established targets of both effectors, suggesting evolution to accommodate the substrates earlier in the evolutionary timeline in a common ancestor of SopD and SopD2. Other Rabs have demonstrated specificity as targets for SopD2 only, suggesting effector divergence in structure and function after the gene duplication event from a common ancestor. Rab8A and Rab8B are collectively designated as Rab8.

Rab GTPases, important regulatory switches that control diverse aspects of vesicular trafficking (biogenesis, transport, tethering, and fusion of membrane-bound compartments) [63,64], represent the most well-characterized targets of these paralogous effectors. Interestingly, several of the identified interactors of SopD have also been identified as interactors of SopD2, including Rab8A and Rab8B, Rab10, and Rab14 (identified in one biological replicate assay using IP-MS) [58]. However, to date, a contribution to infection has yet to be elucidated. While functional studies have yet to be performed in the context of infection, these in vitro assays suggest that the ability to bind to these host proteins evolved before the duplication and divergence of these effectors (Figure 4), especially considering that both effectors have GAP activity against Rab8 and Rab10 [27]. It would also suggest that the GAP site is an evolutionary conserved feature of these paralogs. Since only SopD2 has GAP activity against Rab29, Rab32, and Rab38, it is suggestive of the divergence of the GTPase binding associated with the GAP mechanism, resulting in new targets that evolved after effector duplication (Figure 4) [27]. 

With respect to other Rab GTPases, since the targeting of Rab7A via the N-terminal region of SopD2 was shown to be specific for SopD2 [28], it is likely that this mechanism evolved after effector duplication (Figure 4). Similarly, since Rab34 was shown to be targeted by SopD2 but not SopD [59], this unknown mechanism may have also evolved after effector duplication (Figure 4).

To date, two distinct mechanisms of Rab GTPase targeting have been established for the paralog effectors: GAP activity via a C-terminal arginine for SopD and SopD2, and GTPase suppression by the N-terminal region observed only in SopD2 [28,58]. The former mechanism is characterized by a preferential binding to the GTP-bound Rab [58], and the latter is associated with the absence of a nucleotide-binding preference [28]. For SopD2′s target Rab34, the mechanism of action has yet to be elucidated [59]. SopD2 was shown to preferentially bind the GTP-locked variant of Rab34 via size-exclusion chromatography, which is more consistent with the GAP mechanism. However, in vitro GAP studies have not been performed [59]. Considering that GAP hydrolysis is not the only known mechanism of GTPase targeting associated with preferential interaction with GTP-bound Rab GTPases [63], it is possible that targeting occurs via an alternate mechanism.

While SopD2 has been established to function via a non-GAP mechanism [28], it is also possible that SopD may have evolved a non-GAP mechanism of GTPase targeting. Indeed, SopD’s mechanism of targeting Rab8A has been shown to be more complex than anticipated, as GAP activity was observed to occur in concert with GDI displacement [60]. Similarly, Rab14 and Rab3D were recently identified as interactors for SopD by yeast two-hybrid assay, but SopD did not demonstrate in vitro GAP activity against the GTPases (Table 1) [27]. While it is possible that there is no physiological consequence in the context of infection, it is also possible that an alternate mechanism of manipulation exists. 

Conversely, several Rabs have been shown to be GAP substrates for SopD2 but have not been fully characterized (Rab8A, Rab10, Rab29, Rab38) (Table 1) [27]. In the future, it would be valuable to investigate the possibility of a contribution to infection.

From a broader perspective, Rab GTPases represent a sublineage of the larger Ras superfamily of regulatory GTPases, a structurally similar group of proteins that function via an evolutionarily conserved cycling mechanism [79,80]. Given that the spectrum of Rab targets of SopD and SopD2 is broadly distributed among the Rab phylogenetic tree (Figure 2), it would be interesting to see whether either of these bacterial effectors can target other non-Rab GTPases in the larger Ras superfamily.

SopD and SopD2 display very little overlap in their non-Rab host protein interactors, which could be reflective of either their distinct intracellular localizations [26] or differences in three-dimensional structures that could mediate distinct binding capabilities [28]. While a physiological consequence on infection is yet to be elucidated for most of the hits, if true, it raises the questions of whether the interactions are direct, and if so, whether the domains in the bacterial effectors responsible for binding the host proteins are distinct from those that mediate Rab binding. Considering the structural divergence observed in the N-termini of SopD and SopD2 (Figure 1B) [28], it is also interesting to speculate whether this region might contribute to the differences in host protein interactors. 

SopD and SopD2 play critical roles in infection, as evidenced by their described activities and their contributions to replication and virulence, with SopD promoting early responses to inflammation [60] and bacterial invasion [26,29] and SopD2 contributing to the later stages of the bacterial intracellular life cycle via maintaining SCV stability [16,45], intracellular positioning [15], and modulating endosomal trafficking [28]. Based on the current literature, it is not fully clear whether SopD can protect against the effects of a *sopD2* deleterious mutation, and vice versa. In the case of SopD2-mediated inhibition of Rab7, it was shown that the expression of SopD could not functionally complement a defect of a *sopD2* knockout background [28]. In the context of infection, given that each effector has evolved distinct kinetics of secretion, it is possible that functional complementation may not always apply. Future studies will provide valuable insights in this regard. With respect to expression, it is valuable to note that SopD can also be translocated by the SPI-2 T3SS in vitro [25] and has been detected at later stages of infection in mouse models [81]. Yet, research to date has been limited to SPI-1-related secretion and early timepoints in infection [26,29,60]. Future studies on its potential contributions to later stages of intracellular infection would provide valuable insights. 

**Figure 5:**
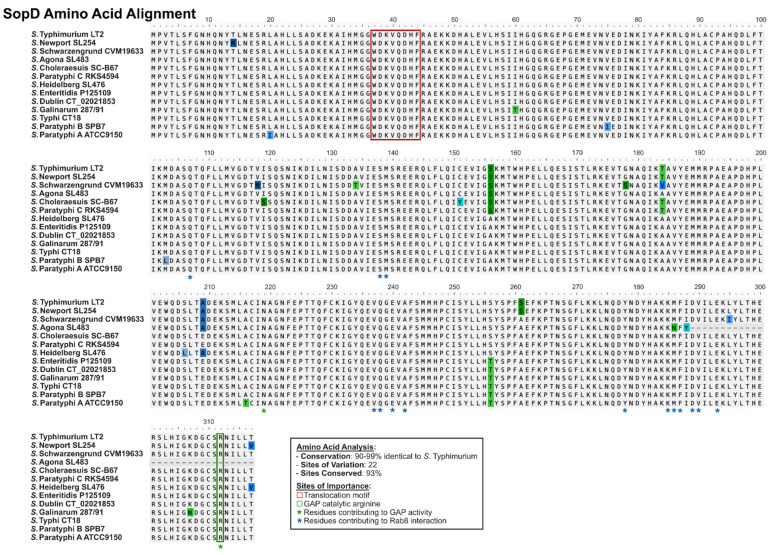
**SopD is highly conserved across *S. enterica* serovars.** An amino acid alignment of SopD from diverse *S. enterica* serovars classified as either predominantly gastrointestinal (i.e., Typhimurium (Accession NP_461866.1), Newport (Genome Accession NC_011080.1), Schwarzengrund (Genome Accession NC_011094.1), Agona (Genome Accession NC_011149.1), Heidelberg (Genome Accession NC_011083.1), Enteritidis (Genome Accession NC_011294.1), and Paratyphi B (Genome Accession NC_010102.1)) or extraintestinal (Choleraesuis (Genome Accession NC_006905.1), Dublin (Genome Accession NC_011205.1), Gallinarum (Genome Accession NC_011274.1), Paratyphi C (Genome Accession NC_012125.1), Paratyphi A (Genome Accession NC_006511.1), and Typhi (Genome Accession NC_003198.1)) [18,42] is shown, where sites of variation are highlighted. Important sites are indicated either as colored boxes and/or asterisks. Where a unique protein accession number is not specified, the associated genome accession number is shown. Amino acids that are divergent from the consensus sequence are highlighted.

While a great deal of functional insights into SopD and SopD2 have been gained in recent years, as with studies on most *Salmonella* effectors, research to date has been limited to the model serovar for *S. enterica*, *S*. Typhimurium [14,16,21,25,26,28,29,45,60], except the initial characterization of *sopD* in *S*. Dublin [31]. Considering that SopD and SopD2 are found in most *S. enterica* serovars, a deeper analysis of effector function in more divergent serovars could provide insights into any potential functional divergences. For example, SopD is highly conserved across serovars previously analyzed [18,42], and SopD in these serovars displays 98–99% sequence identity relative to the *S*. Typhimurium variant (Figure 5). The translocation motif [56] and all residues denoted as critical for the interaction with Rab8A and GAP activity are conserved [60], except in *S*. Agona. Interestingly, *S*. Agona has a premature stop codon in the *sopD* gene rendering the protein truncated at 288 amino acids, and as such, SopD_Agona_ lacks both the catalytic GAP R312 residue and the E293 residue critical for Rab8A interaction (Figure 5) [60]. While the selective pressures that would generate such a mutation in *S*. Agona are unknown, it would be of value to identify the consequences of this mutation on the functionality of SopD in *S*. Agona. Notably, it has been reported that *S*. Agona can secrete SopD in vitro [82], suggesting that this protein may retain some function in this serovar, independent of the GAP activity. However, the high amino acid conservation of SopD in all other serovars suggests that preserving the amino acid integrity of SopD is beneficial to *S. enterica*. 

**Figure 6:**
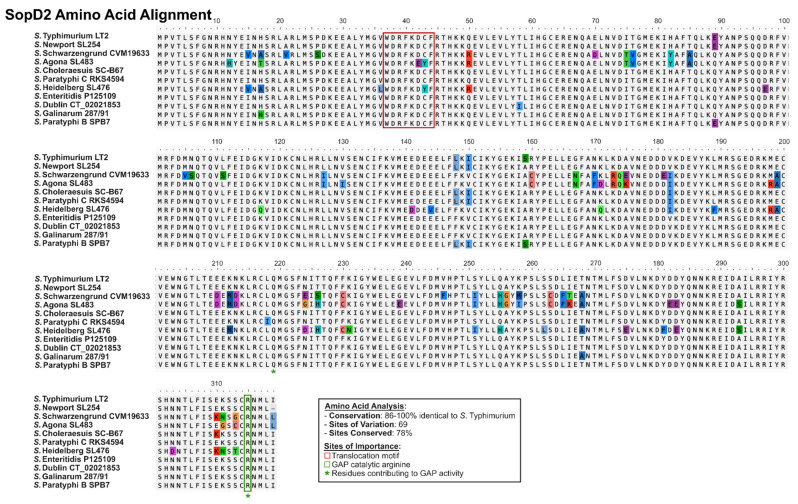
**SopD2 demonstrates more amino acid variability across *S. enterica* serovars than SopD.** An amino acid alignment of SopD2 from diverse *S. enterica* serovars classified as either predominantly gastrointestinal (i.e., Typhimurium (Accession NP_459947.1), Newport (Genome Accession NC_011080.1), Schwarzengrund (Genome Accession NC_011094.1), Agona (Genome Accession NC_011149.1), Heidelberg (Genome Accession NC_011083.1), Enteritidis (Genome Accession NC_011294.1), and Paratyphi B (Genome Accession NC_010102.1)) or extraintestinal (Choleraesuis (Genome Accession NC_006905.1), Dublin (Genome Accession NC_011205.1), Gallinarum (Genome Accession NC_011274.1), and Paratyphi C (Genome Accession NC_012125.1) [18,42] is shown, where sites of variation are highlighted. Important sites are indicated either as colored boxes and/or asterisks. Where a unique protein accession number is not specified, the associated genome accession number is shown. Amino acids that are divergent from the consensus sequence are highlighted.

Conversely, SopD2 displays more amino acid variations across the entirety of the sequence compared to SopD, with 69 variant sites and 27 of them within the first 150 amino acids (Figure 6). Considering that the first 150 amino acids are responsible for interaction with Rab7A, the effects of these site variations on this activity of SopD2 are unknown. Most serovars share 98–100% sequence identity with *S*. Typhimurium, with SopD2 from *S*. Agona, *S*. Schawrzengrund, and *S*. Heidelberg demonstrating the most divergence (86%, 86%, and 89% sequence identity, respectively, relative to the *S*. Typhimurium variant). It is interesting that *S*. Agona also displays the greatest difference in the sequences of SopD2, as was seen with SopD. In particular, the first 150 amino acids of SopD2_Agona_, SopD2_Schawrzengrund_, and SopD2_Heidelberg_ demonstrate the highest levels of sequence divergence (Figure 6). Given that this region includes the N-terminal region of structural dissimilarity with SopD, it would be interesting to speculate whether these strains have evolved additional functions. As such, it would be insightful to characterize the role of SopD2 in these and other more divergent serovars.

In conclusion, SopD and SopD2 are *Salmonella* effectors that play vital yet different roles in the intracellular life cycle of this human pathogen, although they are believed to have descended from a common ancestor. While research to date has uncovered a wealth of knowledge about their mechanisms of action and physiological roles during infection, there is still much to be discovered about these effectors. As such, future studies will provide valuable insights into the genetic and functional divergence of this pair of paralog effectors during the course of evolution. 

## Figures and Tables

**Table 1:** **Known interactors and host targets of the *Salmonella* effectors SopD and** **SopD2.**

Effector	Host Interactors and/or Targets				References
	Interactor (Assay) ^1^	Target ^2^	Mechanism	Infection Phenotype	
SopD	Rab8A (IP-MS, CoIP, Y2H, SEC, GAP)	Yes	C-terminal GAP site ^3^	Upregulation of pro-inflammatory cytokines, downregulation of anti-inflammatory cytokines ^3^	[27,29,60]
	Rab10 (IP-MS, CoIP, Y2H, GAP)	Yes	C-terminal GAP site	Suppression of Rab10, promoting plasma membrane scission (SCV formation)	[27,29]
	Rab8B (Y2H)	ND	---	---	[27]
	Rab14 (Y2H)	ND, NG	---	---	[27]
	Rab3D (Y2H)	ND, NG	---	---	[27]
	VPS35 (IP-MS)	ND	---	---	[29]
	VPS26A (IP-MS)	ND	---	---	[29]
	MAP1B (IP-MS)	ND	---	---	[29]
	CKAP5 (IP-MS)	ND	---	---	[29]
	DCTN2 (IP-MS)	ND	---	---	[29]
	DYNC1H1 (IP-MS)	ND	---	---	[29]
	KIF5B (IP-MS)	ND	---	---	[29]
	KIF11 (IP-MS)	ND	---	---	[29]
	MYO5B (IP-MS)	ND	---	---	[29]
	AP3S1 (IP-MS)	ND	---	---	[29]
	SCYL2 (IP-MS)	ND	---	---	[29]
	CLTC (IP-MS)	ND	---	---	[29]
	AP2M1 (IP-MS)	ND	---	---	[29]
SopD2	Rab7A (IP-MS, CoIP, IVB)	Yes, NG	N-terminal inhibition	Suppression of Rab7A-mediated endocytic trafficking	[28,45]
	Rab32 (CoIP, GAP)	Yes	C-terminal GAP site	Suppression of Rab32-mediated cell- autonomous defense pathway	[27,58,60]
	Rab8A (IP-MS, CoIP, GAP)	Yes	C-terminal GAP site	---	[27,58,61]
	Rab10 (IP-MS, CoIP, GAP)	Yes	C-terminal GAP site	---	[27,29,61]
	Rab8B (CoIP)	ND	---	---	[58]
	Rab34 (IP-MS, SEC, PD)	Yes	---	Rab34, along with SopD2, promotes *Salmonella* replication in epithelial cells	[59]
	Rab29 (GAP)	Yes	C-terminal GAP site	---	[27]
	Rab38 (GAP)	Yes	C-terminal GAP site	---	[27]
	AnxA2 (IP-MS, PD)	Yes	---	---	[62]
	MYH10 (IP-MS)	ND	---	---	[61]
	MYL6 (IP-MS)	ND	---	---	[61]
	MYL12B (IP-MS)	ND	---	---	[61]
	MYH9 (IP-MS)	ND	---	---	[61]
	RBM10 (IP-MS)	ND	---	---	[61]
	EIF3B (IP-MS)	ND	---	---	[61]
	CYFIP1 (IP-MS)	ND	---	---	[61]
	PHB2 (IP-MS)	ND	---	---	[61]
	EIF3A (IP-MS)	ND	---	---	[61]
	AP2B1 (IP-MS)	ND	---	---	[61]
	EIF3E (IP-MS)	ND	---	---	[61]
	AP3D1 (IP-MS)	ND	---	---	[61]
	MYO1B (IP-MS)	ND	---	---	[61]
	AP3B1 (IP-MS)	ND	---	---	[61]
	AP2A1 (IP-MS)	ND	---	---	[61]

^1^ The method of establishing interaction is shown in parentheses. IP-MS: immunoprecipitation–mass spectrometry, CoIP: coimmunoprecipitation, SEC: size exclusion chromatography, PD: pulldown assay, Y2H: yeast two-hybrid assay, IVB: in vitro binding assay, GAP: GTPase-activating protein (GAP) in vitro assay. For mass spectrometry-based assays, hits identified in multiple replicate assays are reported. ^2^ The interactor is defined as a target for the effector if the mechanism of manipulation has been established and/or a consequence on infection has been established. ^3^ The effector also demonstrates guanosine dissociation inhibitor (GDI) activity against Rab8A, resulting in a reversal of the GAP-associated infection phenotypes [60]. ND: not determined, NG: negative result in in vitro GAP assay [28], Rab32 [27,58,60], Rab34 [59], Rab8A and Rab8B [58,60], Rab10 [29,58], Rab29 [27], and Rab38 [27].
